# TOUCH SENSATION IS AN UNDERSTUDIED PREDICTOR OF DEMENTIA RISK IN OLDER ADULTS

**DOI:** 10.1093/geroni/igac059.603

**Published:** 2022-12-20

**Authors:** Willa Brenowitz, Nathaniel Robbins, Elsa Strotmeyer, Kristine Yaffe

**Affiliations:** University of California, San Francisco, San Francisco, California, United States; Geisel School of Medicine, Dartmouth, Lebanon, New Hampshire, United States; School of Public Health, University of Pittsburgh, Pittsburgh, Pennsylvania, United States; University of California, San Francisco, San Francisco, California, United States

## Abstract

Few studies have focused on touch sensation as risk factor or marker of dementia, although other sensory impairments are associated with cognitive decline. We studied touch sensation as measured by peripheral sensory nerve function; impairment was defined as insensitivity to 10-g monofilament or vibration detection threshold >130μm of the toe, in 2,174 Black and White participants (52% women; 35% black, aged 70-79 years) from Health, Aging, and Body Composition Study who were ambulatory and without dementia at enrollment. Incident dementia over the following 11 years was determined based on medical records, cognitive scores, and medications. Impaired touch sensation was associated with a 1.63-fold higher risk of dementia (95% CI 1.21, 2.19) after adjustment for demographics, health behaviors, and health conditions. Associations persisted even after additional adjustment for hearing, vision, and smell (HR: 1.45; 95%CI 1.09, 2.03). These findings highlight the underappreciated association between poor touch sensation and risk of dementia.

